# The Enactment of Knowledge Sharing: The Roles of Psychological Availability and Team Psychological Safety Climate

**DOI:** 10.3389/fpsyg.2020.551366

**Published:** 2020-09-23

**Authors:** Jing Qian, Wei Zhang, Yi Qu, Bin Wang, Meng Chen

**Affiliations:** ^1^Business School, Beijing Normal University, Beijing, China; ^2^School of Government, Beijing Normal University, Beijing, China; ^3^School of Management, Shanghai University, Shanghai, China

**Keywords:** coaching behavior, knowledge sharing, psychological availability, team psychological safety climate, proactive motivation model

## Abstract

Scholars have made great efforts to investigate the antecedents of knowledge sharing. In the current study, we applied the proactive motivation model (Parker et al., 2010) to propose a theoretical model to advance this research line and examined the relationship between coaching and knowledge sharing. A total of 197 subordinates embedded in 32 teams from a logistics company completed the survey questionnaire. Our results show that leaders’ coaching behavior is positively related to employees’ knowledge sharing behavior through increased psychological availability. Furthermore, our results show that the team psychological safety climate can strengthen the effect of psychological availability on employees’ knowledge sharing behavior, as well as the indirect effect of leaders’ coaching behavior on employees’ knowledge sharing via psychological availability (i.e., a moderated mediation effect).

## Introduction

The performance challenges inherent in the current work environment call for sharing and collaboration among team members (De Long and Fahey, 2000; Reinholt et al., 2011; Kim and Yun, 2015). Accordingly, literature from both practitioners and academics highlights the importance of knowledge sharing (e.g., Reinholt et al., 2011; Kim and Yun, 2015). Knowledge sharing refers to the provision of task-related information and experiences to help others as well as the collaboration with coworkers and supervisors to accomplish tasks, develop new ideas, or implement policies or procedures (Cummings, 2004). Previous studies suggest that knowledge sharing is a self-initiated behavior that benefits work teams and colleagues, but it is not a compulsory requirement for employees (Gagné, 2009; Belschak and Den Hartog, 2010). Moreover, knowledge sharing entails substantial costs and risks on the part of the sharer, who may lose competitive advantages through sharing (Kim et al., 2017). As a result, it is not easy to motivate employees to share knowledge with colleagues voluntarily.

Considering the benefits of knowledge sharing for colleagues and teams as well as its potential costs to sharers, scholars have explored various factors that foster employees’ knowledge sharing from different perspectives (Wang and Noe, 2010). In particular, leaders’ behaviors, such as the empowerment of leadership (Srivastava et al., 2006), ethical leadership (Bavik et al., 2018), and respectful leadership (Gerpott et al., 2020), have been highlighted as important factors in facilitating employee knowledge sharing. In this vein, coaching behavior, which refers to a one-on-one process of guiding and encouraging employees to maximize their career potential and improve their work performance (Redshaw, 2000), may also play a significant role in the knowledge sharing process. Previous studies have suggested that coaching behavior would not only exert a positive effect on employees’ performance and work attitudes (e.g., job satisfaction and organizational commitment, Mink et al., 1993; Ellinger et al., 2003; Kim, 2014), but that it would also help build better workplace relationships (Kim and Kuo, 2015). Thus, it is reasonable to propose that coaching behavior might enhance knowledge sharing, which requires good interpersonal interaction.

To better understand how coaching behavior influences employees’ knowledge sharing, we further examine psychological availability as the transformation mechanism under this relationship. Psychological availability is defined as “the sense of having the physical, emotional, or psychological resources to personally engage at a particular moment” (Kahn, 1990, p. 714) and is a reflection of an individuals’ resource level (May et al., 2004). The proactive motivation model (Parker et al., 2010) suggests that contextual variables, such as a leader’s behavior, influence proactive motivation states, thereby promoting or inhibiting employees’ proactive or voluntary behavior. Specifically, the motivation states include *can do* (i.e., Can I do it? Is it feasible?), *reason to* (i.e., Do I want to do this? Why should I act?), and *energized to* (i.e., possesses positive affect). Based on this model, we argue that through coaching behavior, managers can provide support and give confidence to employees, promoting their employees’ psychological availability (that is manifested as *can do* and *energized to* motivation), which in turn increases their knowledge sharing behavior. In other words, when an employee perceives the support and resources provided by a coaching leader, his or her belief in the capacity to accomplish work-related tasks increases. Accordingly, such an employee is more likely to share knowledge.

**FIGURE 1:**
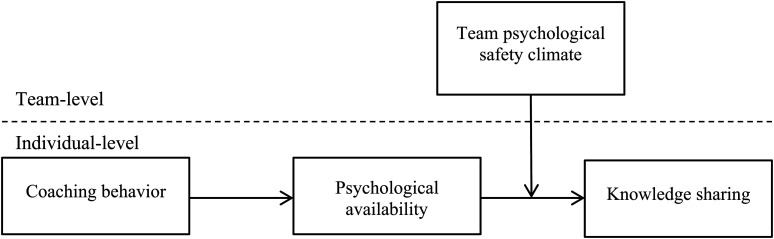
Theoretical model.

In addition, the proactive motivation model suggests that work contexts can influence the effect of an individual’s motivation level (i.e., *can do*, *reason to*, and *energized to*) on being proactive (Parker et al., 2010). We thereby propose that the association between psychological availability and knowledge sharing depends on situational work contexts such as the team psychological safety climate – a shared perception that the work team is a safe environment for interpersonal risk-taking (Edmondson, 1999). The psychological safety of the team encompasses trust, respect, and mutual respect, which is confidence that other members will not disapprove of one’s social interaction (Edmondson, 1996; Koopmann et al., 2016). In line with this, the team psychology safety climate provides employees with a safe, respectful, and trustworthy place to communicate and interact with team members, which helps maximize the positive effect that psychological availability (manifesting as *can do* and *energized to* motivation) exerts on knowledge sharing. In this study, we propose that a high-level team psychological safety climate can strengthen the relations between psychological availability and knowledge sharing. The theoretical model is depicted in Figure 1.

## Theory and Hypotheses

### Coaching Behavior and Knowledge Sharing

Knowledge sharing is regarded as a critical activity for achieving organizational performance and effectiveness in the contemporary knowledge-based economy (Nonaka and Takeuchi, 1995; Kim et al., 2017). However, knowledge sharing is easier said than done. On the one hand, employees are not inclined to share their knowledge because they are afraid of losing key competitive advantages by sharing their valuable knowledge (Cabrera et al., 2006; Masa’deh et al., 2016). On the other hand, sharing knowledge with others can contribute to the occurrence of free-rider problems (Cabrera and Cabrera, 2002). As coaching behavior provides support and gives confidence to employees, we propose a positive relationship between coaching behavior and knowledge sharing based on the proactive motivation model.

Specifically, coaching behavior provides psychological and job-related support for employees’ learning and development (Kim, 2014), fosters employees’ intelligence, and improves employees’ capabilities (Orth et al., 1987). As a result, employees assisted by coaching behavior are apt to form a *can do* (i.e., I can do this) motivation. Coaching behavior is also an efficient way for leaders to guide their subordinates and to create team and organizational cohesion (Orth et al., 1987; Kim, 2014), thereby arousing employees’ *reason to* (i.e., I want to do this) motivation. Moreover, when leaders act as coaches, they spend more time communicating with their subordinates (Hagen and Gavrilova Aguilar, 2012), thus forming high-quality relationships with subordinates and increasing employees’ *energized to* (i.e., I will do this) motivation. Consequently, coaching behavior could serve as an incentive to motivate employees to perform proactive and voluntary behavior (e.g., knowledge sharing). Researchers have noted that motivation is a crucial antecedent to knowledge sharing (Gagné, 2009; Gagné et al., 2019). For example, Gagné et al. (2019) demonstrated that autonomous motivation (i.e., “reason to” motivation) is positively related to knowledge sharing. Accordingly, we propose the following hypothesis:

Hypothesis 1: Coaching behavior is positively related to knowledge sharing.

### The Mediating Effect of Psychological Availability

We suggest that coaching behavior is positively related to psychological availability, which in turn promotes knowledge sharing. Psychological availability represents an individual’s belief that he or she has the physical, emotional, or cognitive resources to engage in work (Kahn, 1990; May et al., 2004). Previous studies suggest that positive social interaction in the workplace could exert positive effects on participants’ psychological state (e.g., psychological availability) and generate desirable outcomes (Collins, 2004; Heaphy and Dutton, 2008). Coaching behaviors, as day-to-day, hands-on processes, give leaders opportunities to positively interact with their subordinates (Orth et al., 1987; Popper and Lipshitz, 1992). When providing coaching behavior, supervisors give guidance, encouragement, and support to the employees (Redshaw, 2000), allowing them to learn easily and quickly with confidence and comfort (Mink et al., 1993). In this process, employees accumulate, manage, and reinforce positive beliefs about their physical, emotional, and cognitive resources. Coaching behavior could, therefore, enhance employees’ psychological availability.

As posited before, knowledge sharing has tangible and intangible costs and risks to the sharers (Kim et al., 2017); thus, one needs to make extra efforts to engage in such behavior. Psychological availability reflects a state where individuals feel capable of directing physical, intellectual, and emotional energies into role performance (Kahn, 1990), and it can help individuals tackle the extra requirements necessitated by knowledge sharing. In this vein, we argue that psychological availability can lead to knowledge sharing for two reasons. First, employees with psychological availability have physical resources to provide help to others (Kahn, 1990) as well as cognitive resources that can generate ideas (Kahn, 1990), leading to *can do* (i.e., possesses the ability to share) motivation. Researchers have demonstrated that “motivation can be understood, at least in part, as the expenditure of resources” (Quinn et al., 2012, p. 339), which provides evidence that physical and cognitive resources may arouse *can do* motivation. Second, employees who have emotional resources (i.e., one dimension for psychological availability) tend to possess self-emotion regulation ability (Kahn, 1990), which helps them form *energized to* motivation, as positive affect is the key element for *energized to* motivation (Parker et al., 2010). In line with the proactive motivation model, we propose that psychological availability allows employees to stay in motivational states, consequently facilitating employees’ knowledge sharing. Accordingly, we propose the following hypotheses:

Hypothesis 2: Coaching behavior is positively related to psychological availability.

Hypothesis 3: Psychological availability is positively related to knowledge sharing.

Hypothesis 4: Psychological availability mediates the relationship between coaching behavior and knowledge sharing.

### The Moderating Effect of Team Psychological Safety Climate

Previous research suggests that a psychological safety climate helps reduce individuals’ defensiveness and encourages employees to focus on collective goals rather than self-protection (Schein, 1993; Edmondson and Lei, 2014). A high-level team psychological safety climate suggests an employee’s high level of trust in team members and a mutual concern for the welfare of those members (Kahn, 1990). In such a climate, employees tend to be motivated to conduct themselves without fear of negative consequences for their core competitiveness (Kahn, 1990); thereby, their felt obstacles to knowledge sharing could be mitigated. In line with this, we argue that the positive relationship between psychological availability and knowledge sharing can be strengthened in a high team psychological safety climate.

In contrast, a low level of psychological safety climate usually indicates negative interpersonal interactions, which manifests as frequent conflict and competition and mistrust between team members (Koopmann et al., 2016). In this climate, workers are more likely to be wary of others and guard their resources (Byrne et al., 2016). As a result, employees in this situation would inevitably be concerned about the several potential costs and risks of knowledge sharing. Some individuals are afraid of losing their competitive advantages (Kim et al., 2017); for example, others might be worried about criticism for sharing incomplete or ill-timed ideas and might feel harmed in the workplace or distracted from their work (Kahn, 1990). For others, it might generate anxiety concerning the occupied resources that could have been otherwise translated into personal engagement in knowledge sharing. Thus, the positive relationship between psychological availability and knowledge sharing would be buffered. Accordingly, we propose the following hypothesis:

Hypothesis 5: The team psychological safety climate will positively moderate the relationship between psychological availability and knowledge sharing in such a way that the relationship will be stronger when the team psychological safety climate is higher rather than lower.

### Overview of the Moderated Mediation Model

Based on the proactive motivation model, we proposed a positive relationship between coaching behavior and knowledge sharing (Hypothesis 1) and argued that psychological availability would mediate the effect of leaders’ coaching behavior on knowledge sharing (Hypotheses 2, 3, and 4). Moreover, as mentioned in Hypothesis 5, the team psychological safety climate is supposed to moderate the relationship between psychological availability and knowledge sharing. In sum, following Edwards and Lambert (2007), these hypotheses and accompanying arguments also indicate the moderated mediation effects. Accordingly, we propose the following hypothesis:

Hypothesis 6: The team psychological safety climate moderates the indirect effect of coaching behavior on knowledge sharing.

## Materials and Methods

### Participants and Procedure

We conducted a survey of 32 workgroups (224 employees and their immediate supervisors) from a logistics company located in northern China. With the assistance of human resource managers, we distributed questionnaires to supervisors and subordinates without giving any specific rewards. Participants completed the survey questionnaire voluntarily, and their responses were matched with identification numbers. Respondents were assured that their responses were confidential. Each participant sealed his or her questionnaire in an envelope provided and returned it 2 weeks later via a secure box outside a company meeting venue.

We received 197 complete ratings of subordinates and their 32 immediate supervisors in the final sample, representing a final response rate of 87.9%. The number of subordinates in each group ranged from 3 to 7 in the final sample, and each supervisor managed 6 subordinates on average. Of the 197 subordinates, 63.5% are men (SD = 0.48), and the average age of the participants was 29.13 years (SD = 5.28). Of the respondents, 23.9% hold a junior high school degree, 21.3% hold a senior high school degree, 18% hold a junior college degree, and 34.5% hold a bachelor’s degree.

### Measures

All scales were originally developed in English. We followed the translation and back-translation procedure recommended by Brislin (1980) to ensure the equivalence of meaning. For all the items, we used seven-point scales (1 = strongly disagree to 7 = strongly agree) to reduce potential central tendency bias in responses.

#### Coaching Behavior

We used a 10-item scale developed by Heslin et al. (2006) to measure supervisors’ coaching behavior. Sample items are “My supervisor supports me in taking on new challenges” and “My supervisor encourages me to continuously develop and improve.” The Cronbach’s α for this scale was 0.92.

#### Psychological Availability

We used a seven-item scale developed by Byrne et al. (2016) to measure employees’ psychological availability. Sample items are “I am emotionally ready to deal with the demands of my work” and “I have the emotional resources to personally invest myself in my work role.” The Cronbach’s α for this scale was 0.81.

#### Knowledge Sharing

We used an eight-item scale developed by Lu et al. (2006) to measure knowledge sharing. Sample items are “I share with others useful work experience and know-how” and “In the workplace, I take out my knowledge to share with more people.” The Cronbach’s α for this scale was 0.85.

#### Team Psychological Safety Climate

We used a seven-item scale developed by Edmondson (1999) to measure psychological safety. Employees were asked to rate their perception of psychological safety about their teams. Sample items are “It is safe to take a risk on this team” and “Working with members of this team, my unique skills and talents are valued and utilized.” The Cronbach’s α for this scale was 0.75. In terms of the aggregation, we obtained the ICC (1)^1^ as 0.213, the ICC (2) as 0.619, and the average Rwg as 0.965 for psychological safety as well as a significant between-group variance [*F*(30,2161) = 2.67, *p* < 0.01], implying the feasibility of aggregating psychological safety to a team-level variable (LeBreton and Senter, 2008). Parenthetically, although the ICC (2) < 0.7, according to group dynamics studies, the team psychological safety climate was a kind of “emergent construct” (Cronin et al., 2011) reflecting the consensus of employees’ safety perception; thus, it can be theoretically defined as a group-level construct (Chen and Bliese, 2002).

#### Control Variables

According to previous studies, we take participants’ gender, age, work tenure, and education level as the control variables in the current study. Age and tenure were measured by the number of years. Gender was coded 1 for “male” and 2 for “female.” Education level was coded 1 for “junior high school,” 2 for “senior high school,” 3 for “junior college,” 4 for “undergraduate,” 5 for “Master’s degree,” and 6 for “Ph.D.”

### Analysis Strategy

First, we conducted a confirmatory factor analysis (CFA) using AMOS 22.0 to examine the validity of our measures. Next, considering the nested structure of our data (i.e., employees nested within supervisors), we conducted a multilevel hierarchical regression using HLM 6.08 to test our hypothesized models (i.e., the mediating role of psychological availability and the cross-level moderating role of the team psychological safety climate). Following prior research (e.g., Ilies et al., 2017), we entered individual-level variables with the group mean centered on removing between-group variances and team-level variables with the grand mean centered. Moreover, to provide further support, following Tofighi and MacKinnon (2011), we also examined the mediating effect of psychological availability using product coefficient analysis with RMediation. Finally, following Edwards and Lambert (2007), we tested whether the team psychological safety climate could moderate the indirect effect of coaching behavior on knowledge sharing through psychological availability (i.e., the moderated mediation model).

## Results

### Confirmatory Factor Analysis

Table 1 shows the CFA results. As shown, our four-factor model (coaching behavior, psychological availability, team psychological safety climate, and knowledge sharing) fits well (χ^2^ = 238.82, df = 113; RMSEA = 0.08; CFI = 0.93; NFI = 0.89). Against the baseline model, we also tested a null model and four alternative models, and the results of alternative models showed poorer fits with the data. Thus, the hypothesized four-factor measurement model is a closer fit.

**TABLE 1:** Results of confirmatory factor analyses.

**Model**	**Factors**	**χ ^2^**	**df**	**CFI**	**NFI**	**RMSEA**	**Model comparison test**		
							**Model**	**Δχ^2^**	**Δ*df***
Baseline Model	Four factors	238.82	113	0.93	0.89	0.08	–	–	–
Model 1	Three factors Coaching behavior and psychological availability combined into one factor	581.98	116	0.74	0.71	0.14	1 vs. baseline	343.16***	3
Model 2	Three factors Coaching behavior and team psychological safety climate combined into one factor	492.40	116	0.79	0.75	0.13	2 vs. baseline	253.58***	3
Model 3	Three factors Psychological safety and psychological availability combined into one factor	496.19	116	0.79	0.75	0.13	3 vs. baseline	257.37***	3
Model 4	Two factors Coaching behavior, psychological safety and psychological availability combined into one factor	834.47	118	0.61	0.58	0.18	4 vs. baseline	595.65***	5
Null Model	One factor	1969.63	153				Null vs. baseline	1730.81***	40

*N* = 197 in 32 groups. CFI, comparative fit index; NFI, normed fit index; RMSEA, root mean square error of approximation. ***p < 0.001.

### Descriptive Statistics

Table 2 shows the means, standard deviations, and bivariate correlations of the study variables. As shown in the table, coaching behavior was positively correlated with knowledge sharing (*r* = 0.25, *p* < 0.01), providing preliminary support for Hypothesis 1. In addition, coaching behavior was positively correlated with psychological availability (*r* = 0.29, *p* < 0.01), and psychological availability was positively correlated with knowledge sharing (*r* = 0.47, *p* < 0.01), which also provides preliminary support for Hypotheses 2 and 3.

**TABLE 2:** Means, standard deviations, reliabilities, and correlations among study variables.

	**Mean**	**SD**	**1**	**2**	**3**	**4**	**5**	**6**	**7**	**8**
1 Gender	−	−								
2 Age	29.14	5.38	–0.006							
3 Education	2.69	1.22	−0.394***	–0.013						
4 Tenure	3.11	4.42	–0.069	0.556***	0.447***					
5 Coaching behavior	5.34	0.81	–0.113	–0.020	0.037	–0.099	(0.92)			
6 Psychological safety	3.71	0.96	0.105	–0.051	−0.190**	–0.072	0.079	(0.75)		
7 Psychological availability	5.32	0.82	–0.125	–0.102	0.078	–0.117	0.293***	0.009	(0.81)	
8 Knowledge sharing	5.39	0.81	–0.040	0.045	–0.069	–0.101	0.251***	0.191**	0.468***	(0.85)

*N* = 197 in 32 groups. Cronbach’s alpha reliabilities are in parentheses on the diagonal. **p < 0.01; ***p < 0.001.

### Mediating Effect Tests

Table 3 showed the results of the regression analyses. For Hypothesis 1, which depicted the direct relationship between coaching and knowledge sharing, the results showed that coaching behavior was positively related to knowledge sharing (*b* = 0.24, SE = 0.09, *p* < 0.05, Model 2); thus, Hypothesis 1 was supported.

**TABLE 3:** Results of regression analysis for mediation and moderation.

**Independent variables**	**Psychological availability**		**Knowledge sharing**					
	**Model 1**		**Model 2**		**Model 3**		**Model 4**	
	***b***	**SE**	***b***	**SE**	**b**	**SE**	**b**	**SE**
Intercept	5.38***	0.56	5.30***	0.75	5.45***	0.62	5.16***	0.60
**Level 1**								
Gender	–0.13	0.11	–0.14	0.17	–0.06	0.14	–0.03	0.15
Age	–0.00	0.01	0.02	0.01	0.01	0.01	0.01	0.01
Education level	0.08	0.10	–0.05	0.10	–0.07	0.08	–0.02	0.08
Tenure	–0.03	0.02	–0.02	0.02	–0.01	0.02	–0.01	0.02
Coaching behavior	0.25*	0.10	0.24*	0.09	0.15*	0.07	0.15^†^	0.09
Psychological availability					0.50***	0.11	0.42***	0.10
**Level 2**								
Team psychological safety climate							0.03	0.11
**Cross interaction**								
Psychological availability (Team psychological safety climate							0.32**	0.14
Level 1 variance	0.56		0.41		0.37		0.37	
Level 2 variance	0.02		0.03		0.07		0.06	
Model deviance	479.25		481.81		442.09		420.91	

*N* = 197 in 32 groups. *p < 0.05; **p < 0.01; ***p < 0.001. Level 2 variance was intercept variance. Model deviance was calculated based on 2 × log likelihood of the full maximum-likelihood estimate, implying the degree of model fitting. ^†^*p* < 0.10.

Hypotheses 2–4 involved the mediating effect of psychological availability on the relationship between coaching behavior and knowledge sharing. As Table 3 showed, coaching behavior was positively related to psychological availability (*b* = 0.25, SE = 0.10, *p* < 0.05, Model 1) and psychological availability was positively related to knowledge sharing (*b* = 0.50, SE = 0.11, *p* < 0.01, Model 3) after controlling for coaching behavior and demographic variables, thus providing support for Hypotheses 2 and 3 and initial support for Hypothesis 4.

To further examine the mediating effect of psychological availability, we used the product coefficient method (Tofighi and MacKinnon, 2011) to test the indirect effect; the results showed that the indirect effect of psychological availability linking coaching behavior and knowledge sharing was 0.125 (SE = 0.058, 95% CI = [0.038, 0.228]). Therefore, Hypothesis 4 was fully supported.

### Moderating Effect Tests

**FIGURE 2:**
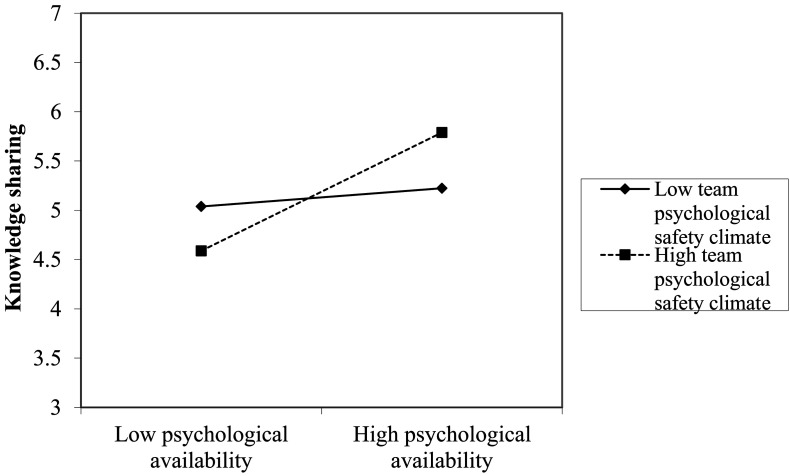
Moderating effect of the team psychological safety climate.

Hypothesis 5 assumed that the team psychological safety climate would positively moderate the relationship between psychological availability and knowledge sharing. To assess the cross-level moderation, we first performed a one-way analysis of variance (ANOVA) for knowledge sharing. Results showed that the ICC (1) was 0.051, indicating that approximately 5% of the variance was contributed by team-level predictors^2^. Then, following Leroy et al. (2015), we obtained a significant between-team level variance for psychological availability to knowledge sharing slope (τ = 0.12, *p* < 0.01). The two points above provided support for the cross-level analysis. As shown in Model 4, the coefficient of the interaction term (i.e., psychological availability × team psychological safety climate) was significant (*b* = 0.32, SE = 0.14, *p* < 0.01, Model 4), providing support for the hypothesis. Following Aiken and West (1991), we also plotted the moderating effect in Figure 2, which showed that the relationship between psychological availability and knowledge sharing was significant and positive (simple slope = 0.73, *p* < 0.01) when the team psychological safety climate was high and was not significant (simple slope = 0.11, ns.) when the team psychological safety climate is low.

Hypothesis 6 depicted the moderated mediation effect of the team psychological safety climate. To test the moderated mediation model, we examined whether the indirect effect of coaching behavior on knowledge sharing through psychological availability differed significantly at different levels of team psychological safety climates (Edwards and Lambert, 2007). Specifically, we estimated the indirect effect of coaching behavior on knowledge sharing through psychological availability at higher (+1 SD) and lower (−1 SD) levels of team psychological safety climates. The results showed that the indirect effect was 0.183 (SE = 0.05, *p* < 0.01, 95% CI = [0.077, 0.288]) when the team psychological safety climate was high and the indirect effect was 0.062 (SE = 0.04, ns., 95% CI = [−0.006, 0.131]) when the team psychological safety climate was low. The estimate of the difference between the two indirect effects was 0.120 (SE = 0.05, *p* < 0.05, 90% CI = [0.028, 0.213]), which indicated that the team psychological safety climate strengthened the indirect effect of coaching behavior on knowledge sharing, providing support for Hypothesis 6.

## Discussion

In the current study, we address an important yet understudied question in the knowledge sharing literature about the role of leader coaching in fostering a focal employee’s knowledge sharing activities. Using a sample of 197 subordinates embedded in 32 teams from a logistics company, our results showed that psychological availability mediated the indirect effects of the leader’s coaching behavior on the focal employee’s knowledge sharing. Additionally, the team psychological safety climate strengthened the relationship between psychological availability and knowledge sharing, and it also strengthened the indirect effect of a leader’s coaching behavior on knowledge sharing through psychological availability (i.e., a moderated mediation effect).

### Theoretical Implications

The present research makes several contributions to the existing body of literature. First, we extend the knowledge sharing literature by identifying coaching behavior as a potential antecedent of knowledge sharing. Previous research has explored the role of individual personality (e.g., Wang et al., 2014; Kim et al., 2017), interpersonal trust and justice (e.g., Wu et al., 2007), and perceived coworker support and cooperative organizational culture (e.g., Swift and Virick, 2013) in predicting knowledge sharing; however, only a handful of scholars have highlighted the importance of leaders’ roles (e.g., Srivastava et al., 2006; Masa’deh et al., 2016). Previous studies have suggested that coaching could benefit employees in terms of their learning and development as well (e.g., Mink et al., 1993; Redshaw, 2000; Ellinger et al., 2003). The present study extends this line of research by identifying coaching behaviors as an important antecedent for knowledge sharing. Therefore, the evidence we provide sheds additional light on the role of leaders in influencing knowledge sharing and supports the positive impact of coaching in a work context.

Second, drawing from the proactive motivation model, the current study provides a new theoretical perspective to explain why contextual factors could exert influence on one’s knowledge sharing. Specifically, contextual factors such as coaching behavior can serve as incentives, that is, providing employees with *can do*, *reason to*, and *energized to* motivation (embedded in one’s psychological availability), to facilitate employees’ knowledge sharing behavior. Although previous studies have applied social exchange theory (Kim et al., 2017), social dilemma theory (Cabrera and Cabrera, 2002), and self-determination theory (Gagné, 2009) to explain the mechanism of knowledge sharing from various perspectives, the role that *can do*, *reason to*, and *energized to* motivations may play in this process remains unknown. These motivations can be obtained by an accumulation of physical, psychological, and cognitive resources. We demonstrate that coaching behavior promotes employees’ knowledge sharing behavior by increasing their psychological availability, which is made up of one’s physical, psychological, and cognitive resources. This could arouse one’s motivational state and thus could motivate participants in the proactive behavior of knowledge sharing.

Third, applying a moderated mediation framework, we investigated the important influence of the team psychological safety climate as a moderator in the relationship between coaching behavior and knowledge sharing through psychological availability. The team psychological safety climate is commonly regarded as a type of team atmosphere that is beneficial for team members (Leroy et al., 2012). In this study, we posit that psychological safety could help channel leaders’ resources toward employees to benefit the team as a whole. Consistent with these arguments, our findings revealed that when the team psychological safety climate is high, it helps to enhance the effectiveness of coaching in encouraging psychological availability and knowledge sharing.

### Practical Implications

Our findings also provide valuable suggestions for managerial practice. First, although facilitating knowledge sharing is difficult, our findings indicated that it might be possible when employees receive assistance and investment from leaders’ coaching behavior based on the proactive motivation model. Therefore, organizations should promote and train coaching behavior among leaders, and in doing so, this might foster the process of knowledge sharing. For example, organizations could help leaders improve coaching efficacy by focusing on motivation, character building, strategy, and technique efficacies (Myers et al., 2005).

Second, given our findings that psychological availability can serve as a mediator in the coaching–knowledge sharing relation, organizations should consider fostering the improvement of psychological availability. Previous research has noted that when individuals perceive low levels of uncertainty and stress, their psychological availability will be increased (Binyamin and Carmeli, 2010). In this respect, managers should clarify their demands and expectations for followers, such as setting clear goals for work processes and precise performance criteria (Barney et al., 1987; Binyamin and Carmeli, 2010). Employee assistance programs (EAPs) or other support resources such as emotion regulation training aimed at helping employees cope with stressors are also important.

Third, our results showed that the mediated relationship was stronger when the team psychological safety climate was higher rather than lower. Our findings indicate that organizations or supervisors must consider the building of a safety team climate when providing coaching benefits. They could enhance the team psychological safety climate by flattening hierarchical differences, improving familiarity among workers, minimizing geographic dispersion, and inviting input as well as opinions from all members (Jain et al., 2016).

### Limitations

Except for the theoretical and practical implications, the current study also has several limitations. First, the survey data were collected at the same time point, which may lead to a common method variance issue and prevent us from establishing causal relationships among the variables. Therefore, time-lagged, longitudinal studies are recommended.

Second, the findings are, to a certain extent, context-dependent because this study was conducted in one company in China. We focused on individual-level knowledge sharing behavior, which is subject to variance in all organizations and cultures (Triandis and Suh, 2002). Likewise, the psychological safety climate might be influenced by cultural factors. For example, research has demonstrated that the psychological safety climate has different influences on risk-taking behavior under team individualism/collectivism contexts (Deng et al., 2019). Therefore, we encourage more research on our hypothesized moderated mediation model in other work settings as well as in other countries by taking cultural differences into account.

Third, considering that we only propose one possible pathway (i.e., psychological availability) to explain how coaching behavior leads to knowledge sharing, a future study could explore other possible mediators from a different theoretical perspective. For instance, based on social cognitive theory, employees’ knowledge sharing ability could also explain the mechanism between coaching and knowledge sharing. Previous research suggested that one of the reasons for inhibited knowledge sharing was the sharers’ fear of providing inappropriate knowledge (Wang and Noe, 2010). Put another way, employees thought that they were unable to share knowledge properly. Given that coaching behavior encompasses one-on-one guidance, facilitation, and inspiration for employees (Heslin et al., 2006), leaders could serve as a role model in this process (Zhou, 2003), increasing employees’ sharing abilities and in turn enhancing knowledge sharing behavior.

Fourth, because coaching, psychological availability, and knowledge sharing are possibly affected by other contextual factors as well as the characteristics of employees, future research could benefit by integrating more moderators to examine the boundary conditions of the coaching influence. For example, future research could include learning goal orientation (Dragoni et al., 2009) as a moderator of the relationship between coaching behavior and knowledge sharing. It may be easier for individuals with a higher learning goal orientation to accept coaching activities, as they are more willing to improve themselves and learn from others, which is beneficial to knowledge sharing.

## Conclusion

The current study extends the knowledge sharing literature by identifying the role of leader coaching behavior in promoting employee knowledge sharing behavior. To further explain this mechanism, we employed psychological availability as the mediator. Our findings reveal that leaders’ coaching behavior can enhance employees’ psychological availability, thereby increasing their knowledge sharing, especially when they work in a team with higher levels of psychological safety.

## Data Availability Statement

The raw data supporting the conclusions of this article will be made available by the authors, without undue reservation.

## Ethics Statement

All procedures performed in studies involving human participants were in accordance with the ethical standards of the institutional and/or national research committee and with the 1964 Helsinki Declaration and its later amendments or comparable ethical standards with written informed consent from all subjects. This research was approved by the Research Committee at the Business School, Beijing Normal University.

## Author Contributions

JQ and WZ substantially contributed to the conception, the design of the work, as well as the preparation of the draft. YQ and BW reviewed it critically and gave important intellectual input. MC contributed to the analysis and interpretation of the data. All authors contributed to the article and approved the submitted version.

## Conflict of Interest

The authors declare that the research was conducted in the absence of any commercial or financial relationships that could be construed as a potential conflict of interest.

## Footnotes

1 ICC (1) = [MSB - MSW]/[MSB + (*k* − 1) × MSW], ICC (2) = [MSB - MSW]/MSB, MSB stands for mean square between groups, MSW stands for mean square within groups, and *k* is the average team size. Rwg (*j*) = *J* × (1 −${\bar{S}}_{X.\tau \,\left(j\right)}^{2}/\sigma_{E}^{2}$)/[*J* × (1 −${\bar{S}}_{X.\tau \,\left(j\right)}^{2}/\sigma_{E}^{2}$) + ${\bar{S}}_{X.\tau \,\left(j\right)}^{2}/\sigma_{E}^{2}$], ${\bar{S}}_{X.\tau \,\left(j\right)}^{2}$ stands for the within-groups variances across the *J* parallel items, and $\sigma_{E}^{2}$ stands for the expected variance when the participant responded randomly (LeBreton and Senter, 2008).

2 According to prior research, an ICC (1) = 0.05 represents a small to “medium” effect, providing prima facie evidence of a group effect (LeBreton and Senter, 2008). We also calculated the Rwg for knowledge sharing; results showed that the average Rwg of knowledge sharing is 0.97, which further certifies the appropriateness for multi-level analysis.
